# Persistently elevated CKMB and negative troponin T in a patient at ischaemic risk with chest pain

**Published:** 2010-02

**Authors:** D Schulenburg, H Van Jaarsveld, JM Kuyl, W de Lange

**Affiliations:** Department of Chemical Pathology, Universitas National Health Laboratory Services, University of the Free State, Bloemfontein, South Africa; Department of Chemical Pathology, Universitas National Health Laboratory Services, University of the Free State, Bloemfontein, South Africa; Department of Chemical Pathology, Universitas National Health Laboratory Services, University of the Free State, Bloemfontein, South Africa; Department of Internal Medicine, University of the Free State, Bloemfontein, South Africa

**Keywords:** analytical interference, assay interference, macrocreatine kinase, creatine kinase interference

## Abstract

Analytical interference in laboratory assays is not only unpredictable but also an underestimated problem. Not recognising these interferences can lead to misdiagnosis and mismanagement of patients. We present a case of a patient with chest pain and ischaemic risk factors with incongruent biochemical results. These results were discovered to be due to the presence of macro-creatine kinase (macro-CK) in vivo interfering with the CKMB activity assay.

## Case report

A 55-year-old female patient, previously diagnosed with biventricular cardiac failure, was admitted to the high-care ward at Universitas Hospital for optimisation of her anti-failure treatment. The left-sided cardiac failure was due to longstanding, poorly controlled hypertension, while the right-sided cardiac failure was secondary to the left-sided failure and obstructive sleep apnoea.

On day four of the admission she developed severe central chest pain. Her risk factors for ischaemic heart disease included: obesity class III (BMI = 54 kg/m^2^), hypertension, hypercholesterolaemia and obstructive sleep apnoea.

The initial troponin T assay (Roche Elecsys 2010) was negative (< 0.03 ng/ml) but the total creatine kinase (CK) and CKMB activity (Beckman Coulter LX-20) was raised to 403 U/l (26–140 U/l) and 435 U/l (< 24 U/l), respectively, resulting in a CKMB percentage of > 100% (< 6%). The ECG showed no evidence of myocardial ischaemia. Cardiology was consulted and it was decided that the cardiac markers be repeated six, 12 and 24 hours later. Repeat biochemical analysis demonstrated the same incongruent cardiac marker pattern (Table 1).

**Table 1:** BIOCHEMICAL RESULTS DEMONSTRATING THE PERSISTENT NATURE OF THE ELEVATED CKMB FRACTION

	Troponin T (< 0.03 ng/ml)	Total CK (26–140 U/l)	CKMB (< 24 U/l)	% CKMB (< 6%)
Index day	< 0.03	403	435	> 100
Four hours later	< 0.03	393	448	> 100
Following day	< 0.03	376	291	77
Two-week clinic follow up after discharge		308	268	87

The persistent nature of the raised CKMB activity led us to suspect that interference was responsible for these results. Re-analysis of dilutions of the samples resulted in non-linear results. Non-linearity in immunoassays generally points to the presence of assay interference although it does not identify the source. We then treated the specimen with polyethylene glycol (PEG) according to a published operating procedure.^1^

When PEG is added to a sample, proteins are excluded from areas in the sample occupied by PEG, raising their concentration in the remaining sample area until precipitation of the proteins occurs. Although various factors play a role, larger proteins are more prone to precipitation than smaller proteins at a given PEG concentration. Therefore PEG has been used to precipitate immune complexes from serum samples.

**Fig. 1.**
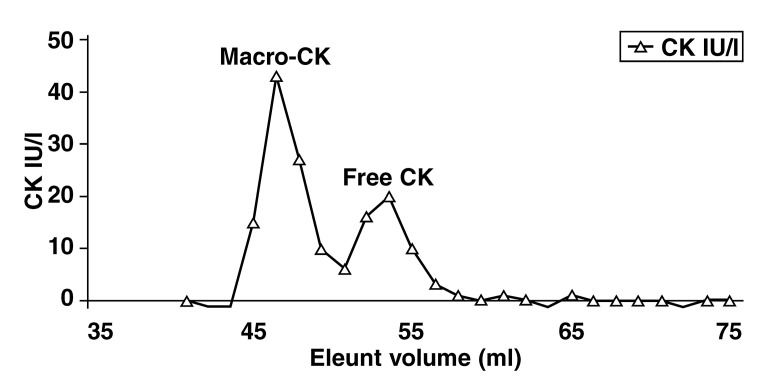
Gel filtration chromatography demonstrating the presence of predominantly macro-CK in the patient specimen. Note that the free CK is well within the normal population reference range.

In our patient’s sample, 60% of the CK activity was precipitated by PEG, which raised the possibility of the presence of macro- CK. As our assay has not been validated for the use of PEG, we used this to increase our index of suspicion only.^1^ We confirmed macro-CK as the interfering compound with the reference method gel filtration chromatography (GFC). GFC is a separation method based on molecular weight (i.e. size exclusion principles). GFC demonstrated that macro-CK contributed 62.7% to the total CK activity and that the free CK (134 U/l) was well within the normal population reference interval (< 167 U/l) (Fig. 1).

## Discussion

Macro-CK is a macro-molecular form of CK that forms and circulates in vivo. The macro-molecules are either CK molecules forming complexes with immunoglobulins (macro-CK type 1) or mitochondrial CK polymers (macro-CK type 2). The most common macro-CK type 1 described is a CK BB-IgG complex. It has been observed in a variety of clinical conditions, as well as in apparently healthy individuals, but no clear correlation has been demonstrated.^2^ Macro-CK type 2, being a mitochondrial polymer, is found predominantly in severely ill patients suffering from malignancies or hepatic disease.^3^ Prevalence is estimated at between 0.5 and 2.6%, but this is influenced by the methodology used and the population selected.^3^

The exact mechanism resulting in CK molecules forming complexes with immunoglobulins remains largely unknown. Due to the high molecular weight of macro-CK, renal clearance is reduced, resulting in an increased half life of the complex.^4^ CK does not have an extracellular catalytic function; so although of no pathological consequence to the patient, the macro-molecule retains enzymatic activity analytically and interferes with total CK and CKMB activity assays, leading to false positive results.^2^

CK catalyzes the reversible phosphorylation of creatine and adenosine triphosphate (ATP) to form creatine phosphate and adenosine diphosphate (ADP). In the routine laboratory, total CK activity is commonly determined by measuring either the forward or reverse products of the reaction.^5^ Several methods are available for the measurement of the CKMB isoenzyme.

In the CKMB immuno-inhibition activity assay, an antibody is used to inhibit the CKM moiety of the CKMB and the CKMM. The resultant CKBB and CKB moiety of the CKMB is then measured similarly to the total CK activity mentioned previously. This method is based on the premise that normally insignificant amounts of CKBB are present in the serum and therefore the CKB moieties measured reflect the amount of CKMB activity in the specimen. It is understandable then that the presence of macro-CK (most commonly a CKBB complex) will falsely elevate the CKMB activity result.

CKMB can also be measured by mass assay. The most common approach to measuring mass is with an immunometric method using antibodies directed against the M or B subunit. Because of the CKMB specificity of the antibodies used, interference poses less of a problem.^5^ A disadvantage is that the mass assay is much more expensive.

An alternative approach to excluding macro-CK interference in a CKMB activity assay would be to have the specimen re-analysed, if available, with a mass assay. When macro-CK interference is suspected in a total CK assay, as requested for the diagnostic workup of muscle disorders or drug side effects, routine method alternatives are unfortunately not available. Screening by PEG precipitation or analysis by CK electrophoresis or gel filtration chromatography remains the only option. These methods are available only in specialised laboratories.

Although cardiac troponin has become the preferred biomarker for cardiac damage, the 2007 American College of Cardiology and American Heart Association guidelines for the management of patients with unstable angina/non-ST-elevation myocardial infarction state that the determination of CKMB remains useful for the diagnosis of early infarct extension and periprocedural infarction.^6^

Although CKMB mass assays are recommended, guidelines published in 2007 by the National Academy of Clinical Biochemistry and the International Federation of Clinical Chemistry state that CKMB activity assays are still an acceptable alternative to mass assays in institutions where mass assays are not available or feasible.^7^

Analytically, troponin immunoassays are also not without problems. False positive results due to macro-troponin, as well as false negative results due to anti-troponin antibodies have been described.^4^

## Conclusion

Although well described, analytical interference due to macrocomplexes remains an underestimated and unrecognised problem. In our experience, clinical awareness is not optimal. Not recognising these interferences can lead to misdiagnosis and mismanagement of patients.

The patient’s chest pain resolved spontaneously and she was discharged. On follow up, the patient has been well, despite the biochemical pattern remaining unchanged.

This case illustrates the importance of good communication between the clinician and the pathologist in optimising patient care.

## References

[1] Fahie-Wilson MN, Burrows S, Lawson GJ, Gordon T, Wong T, Dasgupta B (2007). Prevalence of increased serum creatine kinase activity due to macrocreatine kinase and the experience of screening programmes in district general hospitals.. Ann Clin Biochem.

[2] Rameley AT, Wilding P (1989). Macroenzymes: biochemical characterization, clinical significance and laboratory detection.. Clin Chem.

[3] Panteghini M, Bais r, van Solinge WW (2006:). Enzymes. In: Burtis CA, Ashwood ER, Bruns DE, eds.. Tietz Textbook of Clinical Chemistry and Molecular Diagnostics, 4th edn..

[4] Wu AHB (2006). Cardiac troponin: friend of the cardiac physician, foe to the cardiac patient?. Circulation.

[5] (2003). Clinical Chemistry Theory, Analysis, Correlation, 4th edn.. Mosby,.

[6] (2007). ACC/AHA 2007 Guidelines for the management of patients with unstable angina/non-ST-elevation myocardial infarction – executive summary.. J Am Coll Cardiol.

[7] (2007). National Academy of Clinical Biochemistry and IFCC committee for standardization of markers of cardiac damage laboratory medicine practice guidelines: analytical issues for biochemical markers of acute coronary syndromes.. Circulation.

